# Corticostriatal Hypermetabolism in Moyamoya Disease-Induced Hemichorea: Two Case Reports and a Literature Review

**DOI:** 10.3389/fneur.2021.649014

**Published:** 2021-06-24

**Authors:** Wen-biao Xian, Xiang-song Zhang, Xin-chong Shi, Gan-hua Luo, Chang Yi, Zhong Pei

**Affiliations:** ^1^Department of Neurology, The First Affiliated Hospital, Sun Yat-sen University, Guangzhou, China; ^2^Guangdong Provincial Key Laboratory of Diagnosis and Treatment of Major Neurological Diseases, National Key Clinical Department and Key Discipline of Neurology, Guangzhou, China; ^3^Department of Nuclear Medicine, The First Affiliated Hospital, Sun Yat-sen University, Guangzhou, China

**Keywords:** moyamoya disease, chorea, FDG, PET, basal ganglia

## Abstract

Moyamoya disease (MMD) is a rare cause of chorea, and its pathophysiological mechanism remains unclear. We explore the use of cerebral positron emission tomography (PET) to study brain functional connectivity in 2 patients with MMD-induced hemichorea. Abnormal metabolism of brain was analyzed by ^18^F-fluorodeoxyglucose (^18^F-FDG) PET images. Dopamine transporters (DAT) PET evaluated the integrity of the cerebral dopamine system. A comprehensive systemic literature search of the PubMed database was also conducted. The ^18^F-FDG imaging of our patients showed no responsible hypometabolism in affected brain areas, while hypermetabolism in the affected caudate nucleus, putamen and fronto-parietal areas could be seen. DAT PET imaging was normal in patient 1 (a 23-year-old woman), while remarkably reduced DAT binding was seen in the left striatum of patient 2 (a 48-year-old woman). The literature review of 9 publications revealed that 11 patients who underwent single photon emission computed tomography (SPECT) showed cerebral hypoperfusion in the cortex and subcortical area; ^18^F-FDG PET was performed in 3 cases, which revealed hypermetabolism in the affected striatum in 2 cases. These findings suggest that the striatal and cortical hypermetabolism in the first patient result from underactivity in indirect pathway from basal ganglia-thalamocortical circuits, causing increased activity of excitatory glutamatergic thalamostriatal and thalamocortical projection neurons. The collateral vessels in the basal ganglia might lead to disruption of normal basal ganglia signaling. A dominant left hemisphere with corpus callosal connections to the right basal ganglia resulting into left hemichorea is the most probable explanation for the second patient. We have identified abnormal functional connectivity in basal ganglia-thalamocortical circuits in patients with MMD-induced chorea highlighting the corticostriatal pathway plays an important role in the pathogenesis of MMD-induced chorea.

## Introduction

Moyamoya disease (MMD) is an idiopathic, occlusive, intracranial vasculopathy that is characteristic of progressive stenosis and occlusion of proximal intracranial portion of internal carotid arteries (ICAs) and the main branches within the circle of Willis (1). MMD in children is characterized by recurrent attacks of ischemic stroke, which is contrasting to intracranial hemorrhage in adults (2). Involuntary movement due to chorea is rare as an initial manifestation or during the clinical course in patients with MMD, with an estimated frequency of 3–6% (3), and may complicate the diagnostic considerations. Patients with MMD-induced chorea are reported either in single cases or in a small series (4–7), and its pathophysiological mechanism is poorly understood.

Involuntary movement disorders are generated due to a disruption in the fine balance between excitatory and inhibitory signals connecting the basal ganglia and cerebral cortex. Cerebral metabolic imaging with ^18^F-fluorodeoxyglucose positron emission tomography (^18^F-FDG PET) can be used to detect abnormal excitatory and inhibitory connectivity in the brain, and has been commonly used to obtain specific patterns of regional cerebral glucose metabolism and abnormal functional connectivity in movement disorders including Parkinson's disease (8), multiple system atrophy (9) and corticobasal degeneration (10). However, a limited number of studies have reported the cerebral glucose metabolic patterns in hyperkinetic movement disorders.

Therefore, in this study, we report the clinical profiles of two MMD patients with accompanying hemichorea. Cerebral ^18^F-FDG PET images were specifically studied in these patients, with an aim to detect the abnormal functional connectivity in the basal ganglia pathway. Additionally, we provide a literature review of published case-series of the radionuclide imaging of MMD-induced chorea and compare the results with our study, aiming to unveil the mechanism of chorea in MMD.

## Materials and Methods

### Case Descriptions

#### Patient 1

**Figure 1:**
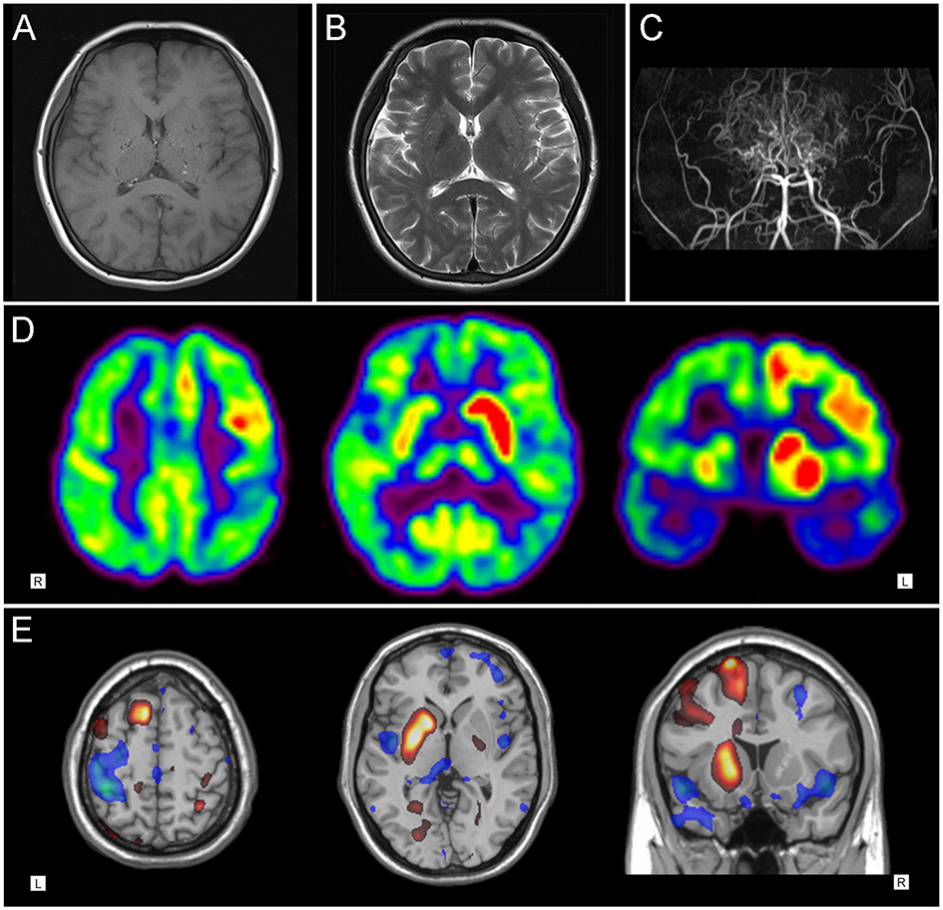
Neuroimaging findings in patient 1. **(A)** T1-weighted and **(B)** T2-weighted axial cranial magnetic resonance imaging showed multiple flow-voids on both basal ganglia. **(C)** Magnetic resonance cerebral angiography revealed severe stenosis in the proximal circle of Willis, with numerous collateral vessels. **(D)** ^18^F-FDG PET images and **(E)** statistical parametric mapping analysis showed hypermetabolism in the left striatum and fronto-parietal cortex, with slight hypometabolism in the left temporal region.

A-23-year-old woman developed involuntary choreoathetoid movements in her right arm, followed by her right leg, which were absent during sleep. On admission, she exhibited choreiform movements of the distal parts of her right arm and leg. Her neurological examination was otherwise normal. Blood tests were conducted to uncover treatable causes of chorea, including erythrocyte sedimentation rate, thyroid function tests, glycosylated hemoglobin A1c, antistreptolysin O antibodies, as well as lupus anticoagulant tests, all of which displayed no abnormality. Brain magnetic resonance imaging (MRI) showed no positive evidence of acute or chronic stroke lesions and multiple flow-voids on both basal ganglia on T1- and T2-weighted images (Figures 1A,B). Magnetic resonance cerebral angiography (MRA) demonstrated severe stenosis in the proximal intracranial portion of the ICAs as well as the main branches within the circle of Willis, with numerous basal collateral arteries being involved (Figure 1C), which conformed to guidelines for diagnosing MMD. Chorea disappeared 3 days after oral administration of haloperidol at a dose of 2 mg/d. Three weeks after admission, left superficial temporal artery-middle cerebral artery (STA-MCA) bypass and encephalo-duro-arterio-myo-synangiosis (EDAMS) were performed. Haloperidol was withdrawn after the operation, and there was no recurrence of choreic movements was reported during follow-up.

#### Patient 2

**Figure 2:**
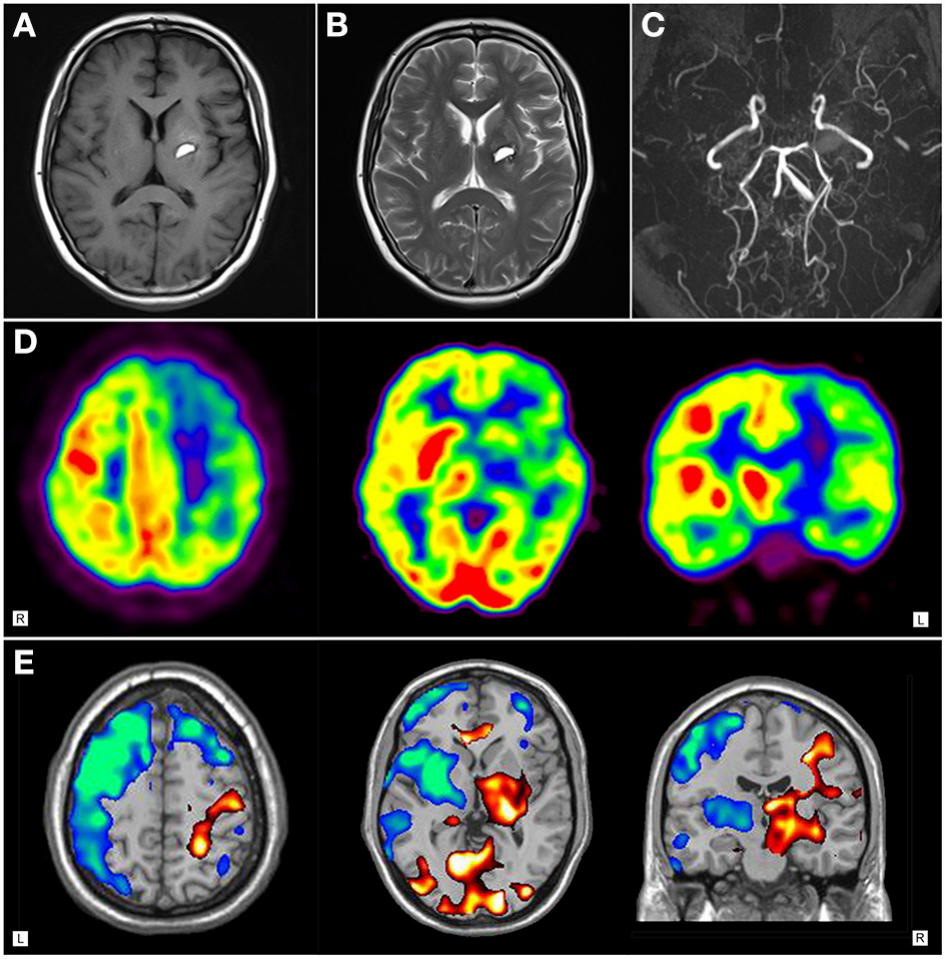
Neuroimaging findings of patient 2. **(A)**T1-weighted axial and **(B)** T2-weighted cranial magnetic resonance imaging showed hyperintense signal in the left basal ganglia. **(C)** Magnetic resonance cerebral angiography revealed severe stenosis of internal carotid arteries in the supraclinoid portion, with numerous collateral vessels. **(D)** ^18^F-FDG PET images and **(E)** statistical parametric mapping analysis showed hypermetabolism in right striatum and fronto-parietal regions, with diffuse hypometabolism in left brain area.

A-48-year-old woman suddenly developed slurred speech and right limb weakness, accompanied with a dull headache. Computerized tomography (CT) showed a hemorrhagic lesion in the left basal ganglia. Four days later, the patient inexplicably developed chorea-like involuntary movements in her left limbs. On admission, the patient presented with facial weakness and sensorimotor hemiparesis on the right side, accompanied with choreic movements involving the left arm and leg. An abnormal Babinski's reflex was observed on the right and brain MRI showed a hyperintense signal in the left basal ganglia on both T1- and T2-weighted images (Figures 2A,B), which was consistent with the diagnosis of intracerebral hemorrhage (ICH). MRA revealed severe stenosis of both ICAs in the supraclinoid portion, with numerous collateral vessels being involved (Figure 2C). The hemichorea movements were gradually alleviated 2 weeks from disease onset, with a full recovery of the right hemiparesis. Two months later, left STA-MCA bypass and EDAMS were performed. No recurrence of involuntary movements was seen during follow-up.

### PET/CT Imaging

Brain PET/CT scans were performed within 1 week after admission (41 days after onset of hemichorea in patient 1, and 9 days after onset of hemichorea in patient 2) when the patients were at the peak stage of symptoms, accompanied with chorea. ^18^F-FDG PET images were acquired with a PET/CT system Gemini GXL 16 (Royal Philips Electronics, The Netherlands) without sedation. Patients fasted for at least 8 h before PET imaging, which started with non-enhanced, low-dose CT imaging about 45 min after intravenous injection of ^18^F-FDG (5.18MBq/kg). PET imaging was performed immediately after CT imaging for about 10 min. Transversal PET slices were reconstructed by means of CT-based attenuation correction using an iterative algorithm. Moreover, individual statistical parametric mapping (SPM) analysis (compared to ten age-matched normal controls, *p* < 0.01; voxel threshold: 50 voxels) was performed to detect the regional glucose metabolism by means of SPM5 (Wellcome Department of Cognitive Neurology, Institute of Neurology, University College London).

The cerebral ^11^C–labeled-2-[beta]-carbomethoxy-3-b-(4-fluorophenyl) tropane (^11^C-CFT) PET imaging of the dopamine transporter (DAT) was also performed on another day for evaluating the integrity of the brain dopamine system.

### Literature Review

A literature review was carried out to identify case series and single case reports regarding chorea related to MMD with PET and single photon emission computed tomography (SPECT) imaging. Cases were identified through a search of the PubMed database utilizing the search strings “moyamoya disease,” “chorea,” “PET,” “SPECT.” Reference lists of identified papers were searched to find additional papers not captured by the initial search strategy. The clinical records, therapies and imaging data were reviewed and summarized in the identified articles. If the information about SPECT or PET imaging was missing or was unclear, cases were not included.

## Results

### ^18^F-FDG PET Imaging

^18^F-FDG PET imaging of patient 1 showed distinct hypermetabolism in the left caudate nucleus, putamen, frontal and parietal cortex, with focally decreased activity in the left posterior-parietal and temporal regions (Figures 1D,E). Patient 2 demonstrated diffuse hypometabolism on the whole left side of the brain. Paradoxically, significantly increased activity was observed in the right caudate nucleus, putamen, frontal and parietal regions (Figures 2D,E).

### ^11^C-CFT PET Imaging

**Figure 3:**
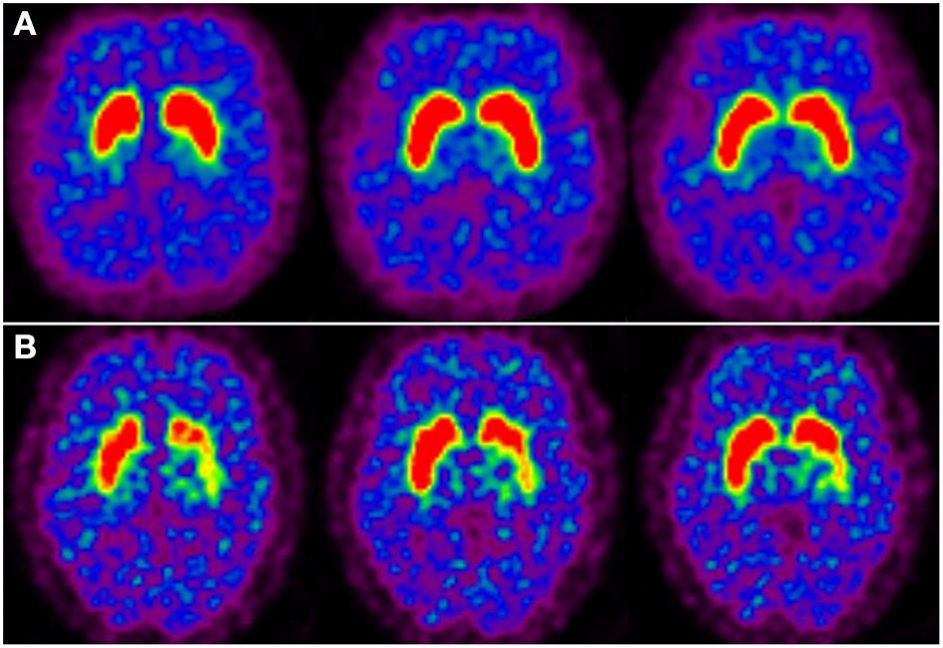
Dopamine transporter PET imaging in moyamoya disease-induced chorea. **(A)** Axail ^11^C-CFT PET images revealed normal bilateral ^11^C-CFT uptake in the striatum of patient 1. **(B)** ^11^C-CFT PET images revealed reduced ^11^C-CFT uptake in the left striatum of patient 2.

^11^C-CFT PET imaging of patient 1 revealed normal ^11^C-CFT uptake in bilateral putamen and caudate nucleus (Figure 3A); while remarkably reduced ^11^C-CFT uptake was seen in the left striatum (especially in the putamen) of patient 2 on PET images, with normal ^11^C-CFT uptake in the right striatum (Figure 3B).

### Summary of the Literature Review

We identified nine publications (6, 7, 11–17) reporting a total of 13 patients that met our inclusion criteria (Table 1). Among the 13 patients identified through the current literature review, the average age at disease onset ranged from 8 to 54 years, and ten of the patients were female. There were nine patients who presented with chorea complicated by MMD as the first manifestation, and ten patients with hemichorea (three patents with involuntary movements in four limbs). Cerebral MRI showed ischemic lesions involving subcortical white matter cortical area in eight cases, small ganglionic infarct in one case, and multiple flow-voids indicating dilated collateral vessels in three cases. Three of the 11 patients that underwent SPECT brain imaging showed a perfusion defect in the basal ganglia, and the remaining eight patients showed cerebral hypoperfusion in the cortex and subcortical area. FDG PET was performed in three cases, which revealed hypermetabolism in the affected stratum in two cases and no obvious changes in one case. Three cases of chorea were eliminated under treatment with medicine, including haloperidol, anticholinergics, and clonazepam. Seven patients underwent STA-MCA bypass, which ameliorated the chorea. However, two patients developed chorea after STA-MCA anastomosis.

**Table 1:** Summary of published case-series of radionuclide imaging of MMD-induced chorea.

**References**	**Number of patients**	**Imaging method**	**CT/MRI findings**	**Radionuclide imaging results**
Hong et al. (11)	1	[99mTc]-HMPAO SPECT	T2-weighted MRI showing multiple small high signal intensity in the left frontal lobe	Perfusion defect in the left basal ganglia
Kim et al. (12)	1	^99m^Tc-ECD SPECT	A previous infarct lesion with extensive encephalomalacia in the left brain	Hypoperfusion within the left brain attributable to a previous infarction
Zheng et al. (13)	1	^123^I-IMP SPECT	T2-weighted MRI showing abnormally high signal lesion in the subcortical white matter	Diffuse cerebral hypoperfusion in the whole brain
Li et al. (14)	1	^99m^Tc-HMPAO SPECT	Multiple dot-like flow voids in the bilateral basal ganglia	Hypoperfusion on the right occipital region
Lyoo et al. (6)	1	^99m^Tc-ECD SPECT	No parenchymal lesion	Hypoperfusion in the right striatum
Pandey et al. (7)	4	^99m^Tc-HMPAO SPECT	Three cases demonstrated small ischemic watershed infarcts involving bilateral frontal subcortical white matter, and one case had a ganglionic infarct	Frontoparietal cortical and subcortical hypoperfusion
Jung et al. (15)	1	^99m^Tc-HMPAO SPECT	Infarct lesion on the right parieto-occipital area and multiple flow-voids in both basal ganglia	Decreased perfusions in the right temporo-occipital cortex and bilateral frontal areas
Sugita et al. (16)	2	^18^F-FDG PET	One case with small asymptomatic ischemic lesion in the right frontal white matter; MRA revealing an dilated and extended lenticulostriate artery in the right striatum in the other case	Reversible hypermetabolism in the affected striatum
Shibata et al. (17)	1	^99m^Tc-ECD SPECT and ^18^F-FDG-PET	No parenchymal lesion	No obvious changes

*^123^I-IMP, N-Isopropyl-p-(I-123) iodoamphetamine; ^99^mTc-ECD, ^99^mTc- ethylcysteine dimer; ^99^mTc-HMPAO, ^99^mTc-hexamethylpropylene amieoxime; ^18^F-FDG, ^18^F-fluorodeoxyglucose; SPECT, single photon emission computed tomography; PET, positron emission tomography*.

## Discussion

Chorea is an uncommon abnormal movement seen in a wide range of disorders, including inherited disorders (18), endocrine diseases (19), and rheumatic diseases (20). MMD has been described as another but rare cause of hemichorea, with striatal hypoperfusion being suggested as the underlying pathophysiological mechanism (7, 21). Therefore, in this study we investigated abnormal functional connectivity of brain in patients with MMD-induced by virtue of ^18^F-FDG PET. Although no hypometabolism was observed in the affected brain areas, significant hypermetabolism was be seen in the affected striatal and fronto-parietal areas. This suggests that the function of the affected striatal and cortical area is activated rather than inhibited in patients with MMD-induced chorea.

As a progressive occlusive disorder of the main cerebral blood supply, the major pathogenesis of MMD is cerebral hypoperfusion. Some studies have reported ischemic lesions in MMD-induced chorea but failed to provide any association between hemichorea and ischemic lesions (7, 22). Cerebral hypoperfusion of the affected areas has been shown in some studies using SPECT in patients with MMD-induced chorea, however this is inconsistent with the abnormalities presented in previous studies found in our literature review. While some studies reported diffuse hypoperfusion of the whole brain area, other studies showed hypoperfusion in the affected stratum. Additionally, cortical and subcortical hypoperfusion in the frontoparietal, temporal, and occipital areas were also reported. The authors suggested that the cortical and subcortical hypoperfusion might interrupt the basal ganglia-thalamocortical circuits, thus resulting in involuntary movements. Of note, the abnormal DAT PET image in patient 2 in our study is probably representing a false-positive finding in the wake of structural abnormality (cerebral hemorrhage). An abnormality on structural imaging may give false-positive results on PET or SPECT imaging as seen with stroke or gliosis. Among the 13 patients identified through the literature review, infarct lesions were shown in MRI in six patients, with decreased perfusions in the affected area in SPECT imaging. Undoubtedly, MMD will lead to decreased cerebral perfusion. However, whether decreased cerebral perfusion is the result of MMD that is caused by insufficient blood supply or the reason leading to chorea, remains unclear from these SPECT studies.

Studies focusing on ^18^F-FDG PET imaging of MMD-induced chorea are rare, and only three cases have been reported so far. For example, it has been reported that striatal hypermetabolism in ^18^F-FDG PET images in two cases with MMD-induced chorea (16), which was in line with our results. Furthermore, we observed increased activity in the affected fronto-parietal cortex of patients with MMD-induced chorea, in accordance with the symptoms of contralateral hemichorea. In our study, one patient presented with the initial manifestation of chorea-like involuntary movements, while the other presented with hemichorea during her clinical course of MMD-induced hemorrhage. Hypermetabolism in the affected striatal and fronto-parietal areas was a common characteristic found in the ^18^F-FDG PET images, although the two patients varied in their clinical manifestations. Meanwhile, cerebral hypometabolism can be seen locally, but it fails to explain the occurrence of chorea. ^11^C-CFT PET scans revealed reduced ^11^C-CFT uptake in the left striatum of one patient, but no changes in ^11^C-CFT uptake in the affected striatum were observed, suggesting that hemichorea has no direct relationship with the dopaminergic neurotransmitter, and instead might be caused by changes in the dopaminergic pathway within basal ganglia-thalamocortical circuits.

**Figure 4:**
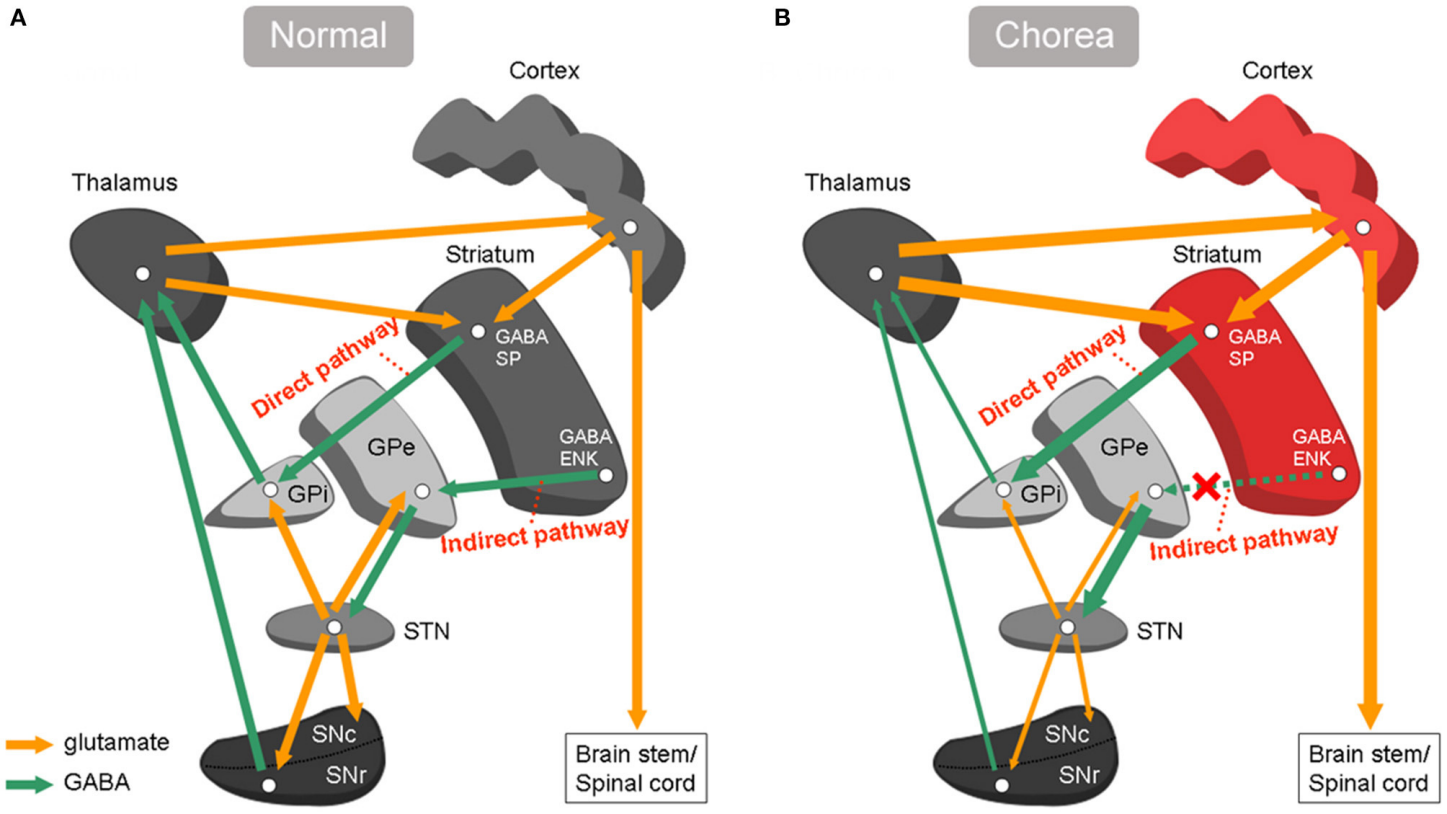
Basal ganglia circuitry under normal conditions and chorea. **(A)** Normal conditions: the striatum projects to output neurons in Gpi through a direct pathway, and by a indirect pathway via GPe and STN. **(B)** Chorea: under-activity in indirect pathway cause increased activity of excitatory glutamatergic thalamostriatal and thalamocortical projection neurons. SP, substance P; ENK, enkephalin; Gpi, globus pallidus internus; Gpe, globus pallidus external; STN, subthalamic nucleus; GABA, γ-aminobutyric acid.

We hypothesize that the striatal and cortical hypermetabolism observed in MMD-induced chorea is due to the disruption of the balance between excitatory and inhibitory signals connecting the basal ganglia and cerebral cortex via the direct and indirect pathways. According to the anatomy of basal ganglia circuits (23), it seems that reduced activity in the indirect pathway from the basal ganglia-thalamocortical motor circuits may be involved in the underlying mechanism of chorea. Reduced activity in the indirect pathway may cause decreased activity of the subthalamopallidal pathway and further decreased inhibitory activity of γ-aminobutyric acid (GABA)-ergic pallidothalamic neurons, which in turn leads to increased activity of excitatory glutamatergic thalamostriatal and thalamocortical projection neurons, leading to increased activity in the affected striatum as well as premotor and motor cortex. This increased activity in the premotor and motor cortex activates excitatory glutamatergic pyramidal tract neurons, finally resulting in chorea (Figures 4A,B). Another explanation could be the result of compensatory changes that occur in the striatum and lead to the eventual resolution of chorea (24). These mechanism may also explain the striatal hypermetabolism in other acute etiologies of chorea, including antiphospholipid syndrome (25), hyperglycemia (26), hyperthyroidism (27), polycythemia vera (28), and Sydenham's chorea (29). In contrast, striatal hypometabolism has been reported in chronic/progressive etiologies of chorea like Huntington's disease (30), spinocerebellar ataxia 17 (31), dentatorubropallidoluysian atrophy (32), McLeod syndrome (33), and chorea-acanthocytosis (34). The observations likely correlate with neuronal loss in theses neurodegenerative diseases. There are cases reports with conflicting evidence in patients with diabetic hemichorea-hemiballism (DHH), who exhibited normal/increased glucose metabolism in the striatum in the acute choreic stage, which progressed to striatal hypometabolism in the later stage after the amelioration of chorea (35). The striatal hypometabolism in the later stage may reflect tissue ischaemia with gliosis in DHH. Thus, striatal hypometabolism in choreic disorders with striatal lesions, either neuronal loss or tissue ischaemia with gliosis, may be correlated with histological changes more than with functional changes relevant to chorea (35). In our study, striatal hypermetabolism was observed in one patient 41 days after onset of hemichorea and the other patient 9 days after onset of hemichorea. Striatal hypermetabolism has been reported in primary antiphospholipid syndrome during the choreic period and also during the non-choreic period 6 months later (36). Striatal hypermetabolism in choreic disorders without striatal lesions correlate with functional changes relevant to chorea, and is often reversible after the amelioration of chorea (16, 25, 28, 29).

In patients with MMD, dilated moyamoya collateral vessels compress the basal ganglia or cause local ischemia, which may further lead to physical disruption of normal striatal signaling and affect the activity of the striatal indirect pathway (23). Additionally, striatal hypoperfusion may also lead to the disruption of normal striatal signaling (6, 15). Interestingly, our patient (patient 2) first developed hemiplegia on the right side caused by left cerebral hemorrhage, with hemichorea involving the left limbs 4 days later. To the best of our knowledge, this is the first case reported in which ipsilateral hemichorea developed following MMD-induced cerebral hemorrhage. The rare condition seems to occur by a different mechanism other than by direct vascular insufficiency of the caudate. A case of ipsilateral hemichorea-hemiballism following ischemic stroke has been described (37). A dominant left hemisphere with corpus callosal connections to the right basal ganglia resulting into left hemichorea is the most probable explanation. There is strong disinhibition of the left thalamus due to the hemorrhage, resulting in strong excitatory input to the ipsilateral cerebral cortex and then to left corticospinal tract. The right hemiplegia suggests that the left corticospinal tract was damaged, so excitatory output was channeled to the opposite (right) motor cortex via the corpus callosum. PET can sensitively detect the increased activity of the afferent corticostriatal pathway in patients with MMD-induced hemichorea. The reason for the inconsistent results between previous SPECT and PET studies is likely because SPECT studies mainly focus on cerebral blood perfusion whereas FDG PET studies evaluate cellular metabolic activity. Therefore, SPECT studies suggest that decreased cerebral blood flow perfusion is the main hemodynamic change caused by MMD, while PET studies have revealed increased metabolism in the motor cortex and basal ganglia as reflected by the activity changes of the corticostriatal network.

In conclusion, our findings indicate that MMD-induced hemichorea results from changes in corticostriatal network activity, causing increased metabolism in the affected cortex and basal ganglia. The limitation of our study is the small sample size. Our patients are adults with MMD and have some features which may be different from children. Our findings offer new imaging evidence of the underlining mechanism of MMD-induced chorea and provide a basis and motivation for future larger scale studies.

## Data Availability Statement

The raw data supporting the conclusions of this article will be made available by the authors, without undue reservation.

## Ethics Statement

The studies involving human participants were reviewed and approved by local Ethical Committee of the First Affiliated Hospital of Sun Yat-sen University. The patients/participants provided their written informed consent to participate in this study.

## Author Contributions

W-bX: conceptualization, data curation and analysis, and writing original draft. X-sZ: data curation and analysis and writing original draft. X-cS, G-hL, and CY: data curation and analysis. ZP: project administration, resources, supervision, and review and editing. All authors contributed to the article and approved the submitted version.

## Conflict of Interest

The authors declare that the research was conducted in the absence of any commercial or financial relationships that could be construed as a potential conflict of interest.
